# Is the HIV Epidemic Stable among MSM in Mexico? HIV Prevalence and Risk Behavior Results from a Nationally Representative Survey among Men Who Have Sex with Men

**DOI:** 10.1371/journal.pone.0072616

**Published:** 2013-09-05

**Authors:** Sergio Bautista-Arredondo, M. Arantxa Colchero, Martín Romero, Carlos J. Conde-Glez, Sandra G. Sosa-Rubí

**Affiliations:** National Institute of Public Health (INSP), Cuernavaca, Mexico; McGill University AIDS Centre, Canada

## Abstract

**Background:**

Recent evidence points to the apparent increase of HIV prevalence among men who have sex with men (MSM) in different settings with concentrated epidemics, including the Latin American region. In 2011, Mexico implemented an ambitious HIV prevention program in all major cities, funded by the Global Fund to Fight Aids, Tuberculosis and Malaria. The program was intended to strengthen the prevention response for the most at risk populations: MSM and injecting drug users. This paper presents the HIV prevalence results of a nationally representative baseline survey in 24 Mexican cities throughout the 5 regions in the country and reports the socio-demographic and sexual risk behaviors that predict the probability of infection.

**Methods:**

The survey was implemented in two phases. We first identified and characterized places where MSM gather in each city and then conducted in a second phase, a seroprevalence survey that included rapid HIV testing and a self-administered questionnaire. The prevalence of HIV was estimated by adjusting for positive predicted value. We applied a probit model to estimate the probability of having a positive result from the HIV test as a function of socio-demographic characteristics and self-reported sexual risk behaviors.

**Results:**

We found an overall HIV prevalence among MSM gathering in meeting points of 16.9% [95% CI: 15.6–18.3], significantly higher than previously reported estimates. Our regression results suggest that the risk of infection increases with age, with the number of sexual partners, and among those who play a receptive sexual role, and the risk decreases with higher education.

**Discussion:**

Our findings suggest a higher HIV prevalence among MSM than previously acknowledged and that a significant regional variability exist throughout the country. These two findings combined, signal an important dynamic in the epidemic that should be better understood and promptly addressed with strong prevention efforts targeted at key populations.

## Introduction

Official reports and UNAIDS estimates have consistently portrayed a stable HIV/AIDS epidemic in the Latin American (LAC) region during the past decade [1]. However, more recent evidence points to the apparent increase of HIV prevalence among men who have sex with men (MSM) in different settings with concentrated epidemics, and evidence from the region points to higher estimates than previously acknowledged [2], [3].

Results from the Western world – mainly from Europe and North America, suggest a significant increase on the HIV prevalence among MSM. According to results from the EuroHIV network, the number of new HIV diagnoses in MSM increased about 100% in 13 Western European countries between 1999 and 2006 [2]. In Amsterdam alone, the incidence of HIV among MSM attending STD clinics was 1.8% in 1998 and increased to 3.8% during the period 1999–2005 [2]. In the United States according to the Centers for Disease Control, during the period of 2001–2006, the incidence of HIV/AIDS among MSM increased 8.6% and the trend was particularly high among black MSM whose incidence reached 12.4% [4].

Very scarce information exists in the LAC region on the HIV prevalence or incidence trends, however the existing data document a highly variable prevalence rate in different countries, between 9.8% and 22.3% [2] and even within countries: a recent study conducted in Brazil, documented a prevalence of HIV in 10 cities of 14.2%, with a significant geographic variation ranging from 5.2% to 23.7% [3].

Consequently, the HIV epidemic continues to be disproportionally high among MSM in concentrated epidemics. A meta-analysis of HIV studies among MSM in low and middle-income countries estimated that globally by 2007 MSM have 19.3 times higher odds of having HIV compared to the general population, while the estimated odds ratio for the Americas was even higher: 33.3 [5].

Among the possible reasons behind this trend, a disinhibition or compensation effect caused by the advent of HAART and its dramatic impact on survival in settings where antiretroviral treatment is widely available, is perhaps one of the most commonly cited. In a study in San Francisco, 48,888 MSM were observed between 1994 and 2002 and a causal link was estimated between the increase in availability of HAART and the increase in sexual risk behavior [6]. Another example is a study that collected data between 2007 and 2008 on HIV/AIDS patients in Mozambique and on a sample of households with no HIV/AIDS patients as controls. The authors found a positive association between perception of ART been efficacious and risk behaviors [7].

However, the lack of access to effective prevention programs [8] and specifically the overwhelming reliance on behavioral change communication (BCC) strategies as the main prevention technology available in many of these settings – particularly in the LAC region might have also played a key role in the expanding epidemics among MSM.

Another very important factor to consider is the lack of comprehensive testing programs and the difficulty for those with a positive diagnosis to initiate antiretroviral treatment. A consequence of these two factors is that a very high proportion of people living with HIV do not know their status, as currently seen among MSM in Mexico [9], which in turn results in high rates of late antiretroviral treatment initiation [10], [11]. Thus, given that treatment as prevention is a well a documented effective prevention mechanism, arguably the best alternative for HIV prevention in concentrated epidemics, it is unavailable for a great proportion of eligible persons [12].

Despite recent calls for attention to this problem, the dearth of epidemiologic and behavioral information to effectively and efficiently design, target and implement prevention programs for these groups is appalling, particularly in the LAC region, and unfortunately Mexico is an illustrative example of this situation.

Ranking third in the Americas in total number of people living with HIV, only behind the US and Brazil, Mexico has a concentrated HIV epidemic. According to UNAIDS, the HIV epidemic in Mexico has remained stable both among the general population and the most at risk populations over the past decade [1]. The HIV prevalence in the country has been consistently reported at around 0.3% among the general population and around 10% among MSM, which constitutes the most affected group [13]. One of the most important sources for this estimated 10% prevalence among MSM, is a survey conducted in 2005 in four cities, with a non- representative sample of 1,111 participants and a response rate of barely 68% [14]. A previous survey in 2003 conducted in a different city estimated a prevalence of 13.9% (n = 399) [15].

While Mexico has been a good example on the treatment front in the Region, granting universal access to antiretroviral treatment since 2003 [16], the same has not been the case on the prevention front. The prevention strategy in the country has been almost exclusively focused on BCC campaigns with very little attention to HIV testing among key populations. According to UNAIDS, HIV prevention efforts in the LAC region require greater attention. The agency has expressed “concerns regarding commitment to HIV prevention” in the region, highlighting, among other things, that “commitment to evidence-informed HIV prevention has been highly variable” and that “only a small fraction of HIV prevention spending in the region supports prevention programs specifically focused on” the most at risk populations, including MSM [17].

In a significant effort to break this inertia of lack of information and poorly designed and unevaluated prevention programs, Mexico applied in 2009 for funding to the Global Fund to Fight Aids, Tuberculosis and Malaria (GFATM) to strengthen the prevention response of the country, with strong focus on the most at risk populations: MSM and injecting drug users. One of the components of this innovative proposal was an impact evaluation of the prevention program. In this paper we present the baseline results of this evaluation, which yielded an unexpectedly disquieting diagnostic of the HIV epidemic in MSM in the country. Specifically, the objective of this study is to present the results of a nationally representative survey conducted in 24 cities (from 5 geographical regions) of Mexico, on HIV prevalence and associated socio-demographic characteristics and self-reported sexual risk behaviors. To our knowledge, this is the first nationally representative survey that includes measurement of HIV among MSM in Mexico, and one of the very few in the LAC region.

## Methods

### HIV Prevention Program

**Figure 1:**
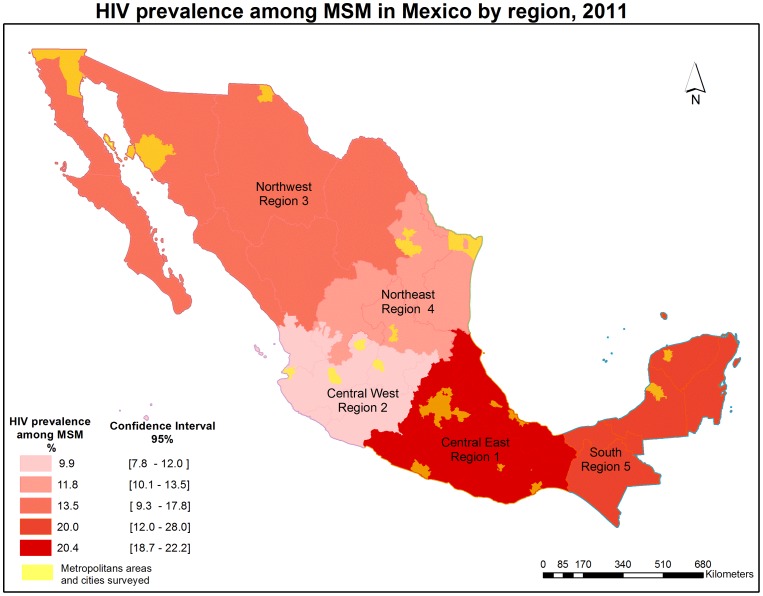
HIV prevalence by rapid testing among MSM in Mexico by region, 2011. Region 1: MA of México City, MA Puebla-Tlaxcala, MA Toluca, MA Cuernavaca, MA Veracruz, MA Acapulco, MA Xalapa, MA Pachuca, MA Tehuantepec-Juchitán; Region 2: MA Guadalajara, MA León-Silao, MA Aguascalientes, Puerto Vallarta; Region 3: MA Tijuana, MA Juárez, Mexicalli, Hermosillo; Region 4: MA Monterrey, MA San Luis Potosí, MA Reynosa-Río Bravo, Matamoros; Region 5: MA Mérida, MA Cancún, Campeche. (MA = metropolitan area).

In 2010, Mexico received funds from the GFATM to implement prevention strategies targeted to the most at risk populations. According to the program objectives, 44 cities were targeted for the prevention strategies' implementation. The 44 cities designated to receive the program, were selected based on size of the cities and prevalence of HIV. According to official estimates, those 44 cities concentrate 72% of the AIDS cases reported among MSM in Mexico in the period of 2003–2008 [18]. Once the cities were identified, they were classified into one of five regions: Central East, Central West, Northeast, Northwest and South (figure 1 displays the list of cities per region). In order to conduct the impact evaluation of the prevention program, a subset of 15 cities were selected to start receiving the program in a first phase (early treatment group – starting in September 2011) and the rest of the cities would start receiving the program six months later (late treatment group).

### Selection of cities for the baseline survey

From the 44 cities, we selected 24 for the impact evaluation and survey implementation. To obtain our sample, the 44 cities were first stratified by program implementation phase (early versus late treatment). Twelve cities were selected from the early treatment group as follows: Mexico City, Puebla-Tlaxcala, Tijuana, Toluca, Mexicali, Cuernavaca, Hermosillo and Veracruz were selected with probability equal to one given population size and their importance for the HIV epidemic in Mexico; the remaining cities were selected with probability proportional to the estimated number of MSM in each city. In the late treatment group, 12 cities were selected as follows: the largest cities such as Guadalajara, Monterrey as well as two touristic cities where there is a large gay community and several meeting points (Cancún and Puerto Vallarta) were selected with probability equal to one; the remaining cities were selected with probability proportional to the estimated number of MSM in each city. The final sample of 24 cities included 12 early treatment which comprised 61% of the final sample and 12 late treatment cities with 39% of the final sample.

Following the selection of cities for the study, we implemented the study in two phases. First, we identified and characterized places where MSM gather and meet sexual partners. In a second phase, we conducted an HIV seroprevalence survey that included a self-administered questionnaire with socio-demographic and risk behavior questions.

### Phase 1: Mapping of MSM gathering places

We adapted the “Places” (Priorities for Local AIDS Control Efforts) methodology to identify the gathering points most frequently mentioned by key informants in each city [19]. In order to identify gathering places, we first collected information from key community informants and constructed a list of the most important and well-known places where, according to their knowledge, MSM tend to meet potential sexual partners. From this initial list of places, which largely consisted of bars and public spaces such as parks, we started the construction of the map of gathering places in each city. Secondly, we went to all places in the initial list and applied a questionnaire to owners/managers or a client only when the former were not available. We interviewed one respondent per site and the information we collected included: size (average number of MSM on the day of maximum attendance), opening hours, days and times of high attendance, proxy measures of socioeconomic status measured by prices of beer and other drinks, feasibility to implement prevention interventions and reference to four additional meeting places where the clients/users of that specific site like to gather as well. These new sites joined the list of gathering places and were visited subsequently to conduct the same process, until the reference network of sites was saturated, i.e. the four sites referred to in new places were already in our list. The complete list and network of references of all gathering places identified through this process constituted the sampling framework for phase 2 of the study.

### Phase 2: Sero-prevalence survey among MSM in gathering places

#### Sample size, measurement, recruitment and ethical considerations

For each of the 24 cities selected, a random sample of gathering places was drawn from the sampling framework constructed through the mapping exercise in phase one. Places with a reported size of less than 9 MSM per night on the day of maximum attendance were excluded from the list. We also excluded sites reportedly not opened to conducting any prevention activities, given that these sites were not relevant for the evaluation of the prevention program. From the 468 sites initially mapped, 80 were excluded because they had a maximum attendance of less than 9 MSM or no information available and 43 sites were excluded because of their unwillingness to conduct prevention activities, leaving 345 from which we randomly selected sites for the survey.

We estimated a sample size of 8,586 individuals sufficient to detect a 50% incidence decrease attributed to the interventions, assuming an HIV incidence rate at baseline of 2%, Assuming an estimated prevalence among MSM as reported by the National HIV Program of 10% [13] and 6 year duration of disease. We used a two stage probabilistic sampling design. We selected the cities, as described previously, in the first stage and the gathering places in the second stage. The gathering places were treated as clusters, characterized by a high intra-class correlation coefficient, i.e., people within any given gathering place are assumed to be more similar than people from other places [20]. The intra-cluster correlation coefficients were obtained from a similar previous survey in Mexico City [21].

From August to November 2011, we conducted an HIV seroprevalence survey that included a self-administered, pre-programed questionnaire in laptops (ACASI: audio computer-assisted self-interview) [22] and a rapid HIV test from standard diagnostics was applied on site to participants (Inc South Korea, HIV ½ LAFON as marketed in Mexico, HIV ½ SD BIOLINE 3.0 elsewhere). This immunochromatographic test was used immediately upon finger pricks following the manufacturer's specified procedure. Sensitivity and specificity of the test are 100% and 99.8% respectively according to the manufacturer and other independent sources [23], [24].

The Institutional Review Board (Ethics Committee) at the National Institute of Public Health in Mexico (IPF Code 3627801) approved the survey. The questionnaire had the following sections: socio-demographic characteristics, health care utilization, sexual risk behaviors and stigma and discrimination.

To avoid enumerators selecting participants based on their own potentially biased criteria, supervisors were in charge of placing each enumerator in a specific spot from which they could easily address people arriving at the gathering places. From their spot, enumerators approached any person who would pass nearby them (excluding women) to invite them to participate in the study. Enumerators explained the objective of the project and what their participation entailed. All men interested in participating were read the informed consent form and if they voluntarily agreed to participate then signed the form. The first module of the questionnaire was designed to screen for MSM. When a non-MSM was identified, as defined by not having had sex with men in the past 12 months, the questionnaire stopped. Non-MSM participants (10% of the sample) were excluded from the analysis and were not counted in the final sample size.

To guarantee confidentiality, names and addresses of the respondents were not requested. Results from the rapid test were not provided to the participants during the survey for two reasons: first, because of the inadequate conditions of most of the gathering places to provide counseling to participants and more importantly to provide appropriate post-counseling for those with an HIV-positive result. Second, given the objectives of the study, the survey was designed to provide a population estimate of the prevalence of HIV, and was not designed to serve as a testing campaign. Moreover, privacy in gathering points such as bars and clubs were impossible to guarantee. However, all participants were provided with printed information containing a list of sites where they could be tested for HIV with pre- and post-counseling for free and with additional information encouraging them to do so. They also received a pack of condoms. In addition, about a month after the survey, the population received several prevention interventions as part of the activities funded by the GFATM including condom distribution, voluntary testing and counseling and other behavioral interventions. The sample of sites where we conducted the survey was prioritized for program implementation.

### Analysis

The prevalence of HIV was estimated by adjusting for positive predicted value of the test using the following parameters: percentage of positive results, 99.8% specificity and 100% sensitivity. Cluster analysis was applied to reflect the sampling design; observations were weighted by the probability that a given city was selected.

We applied a probit model to estimate the probability of having a positive HIV test result as a function of socio-demographic characteristics and self-reported sexual risk behaviors. In our models we included age and age squared to test for non linear associations and educational level completed classified in the following three categories: primary school or less, secondary school, high school and university or higher. We also included self-reported sexual identity: gay, versus other (bisexual, transgender and other). The model also included number of sexual partners in the last month, self-reported condom use in the last sexual intercourse, sexual role in the last sexual intercourse as insertive, receptive or both (the questionnaire asked for active (insertive), passive (receptive) or both in the last sexual intercourse) and geographical region.

From 8,503 individuals who participated in the survey, 7,823 accepted the rapid HIV test. Our analytical sample was reduced to the 6,723 individuals for whom we had information on all covariates. We also excluded those individuals who reported being sex workers (n = 320) because their sexual risk behavior profile was significantly different from the rest of the population. To evaluate any potential bias introduced by missing data, we tested for statistical differences in all variables between the analytical sample and the individuals that either did not have information on all covariates or did not accept the rapid HIV test.

## Results

### Phase 1 results

We identified 468 gathering places from which we were able to collect information on 436 of them. A large percentage of meeting places were discotheques, bars and clubs (43%), saunas (12%), 13% where public places (streets, parks, subway stations), cinemas (6%) and other (26%). According to the informants reckoning, some type of prevention activities, such as condom promotion and pamphlet distribution, would be feasible in 80% of the gathering places; however educational talks and prevention video clips would only be allowed in 40% and 30% of places respectively. We also found that 47% of the meeting places had condoms available on the day of the interview.

### Phase 2 results

From the 468 meeting places identified in the mapping exercise 436 had information available on site and 242 were systematically selected for the survey sample. The survey was completed by 8,503 individuals and 92% of them accepted to provide a capillary blood sample for the HIV measurement.

We found an overall HIV prevalence of 16.9% [95% c.i.: 15.6%–18.3%] (Figure 1). The highest rates were found in Region 1 and 5 with prevalences higher than 19%, followed by Regions 4, 3 and 2 (Table 1).

**Table 1:** Estimated HIV prevalence by region in participant subjects.

Region	N	Prevalence*	Linearized Standard Error	95% Confidence Interval	
Region 1	4,222	20.43%	0.0087	[18.70–22.15]	
Region 2	1,637	9.87%	0.0102	[7.84–11.90]	
Region 3	754	11.84%	0.0085	[10.14–13.52]	
Region 4	762	13.54%	0.0216	[9.27–17.81]	
Region 5	448	19.98%	0.0405	[11.97–27.98]	
**TOTAL**	**7,823**	**16.99%**	**0.0069**	**[15.61–18.37]**	

* *Adjusted for positive predicted value.*

Table 2 shows descriptive statistics of the analytical sample in terms of their socioeconomic background and sexual behavior. The participants were on average 27 years old and had high educational levels relative to the general population in Mexico (42% had completed a university degree or higher). The majority of the participants identified themselves as gay/homosexual (73%). They reported on average 2 sexual partners in the last month; 72% reported using condom in their last sexual intercourse and 33% stated their sexual role was receptive.

**Table 2:** Socioeconomic and risk behavior characteristics of 6,723 study participants.

Variable	Mean/Proportion	Linearized standard error	95% Confidence Interval
Age	27.4	0.243	[26.99–27.95]
Last school grade completed			
Primary school or less	0.04	0.005	[0.03–0.05]
Secondary school	0.13	0.008	[0.12–0.15]
High school	0.41	0.009	[0.39–0.43]
University or higher	0.42	0.013	[0.39–0.42]
Sexual identity (gay = 1, 0 otherwise)	0.73	0.011	[0.71–0.75]
Number of sexual partners (last month)	2.03	0.073	[1.88–2.17]
Condom use (last sexual intercourse)	0.73	0.007	[0.71–0.74]
Sexual role (last sexual intercourse)			
Receptive	0.33	0.007	[0.31–0.34]
Insertive	0.34	0.006	[0.32–0.35]
Insertive & receptive	0.33	0.006	[0.31–0.34]
Region			
Region 1	0.53	0.024	[0.48–0.58]
Region 2	0.16	0.038	[0.08–0.23]
Region 3	0.17	0.041	[0.08–0.25]
Region 4	0.07	0.008	[0.05–0.08]
Region 5	0.06	0.034	[−0.002–0.131]

In Table 3 we present the results of the probit model to estimate predictors of a positive result to the rapid test, controlling for differences across regions. We found that age increases the probability of a positive result but at a decreasing rate. Having completed a university degree or higher is associated with a lower probability of a positive result compared to high school, secondary school or less. As expected, number of sexual partners is a risk factor as well as a receptive sexual role. Unexpectedly, self-reported condom use is associated with a higher risk of being HIV positive.

**Table 3:** Predictors of positive results to HIV rapid testing (probit model^a^) according to selected variables.

Variable	Coefficient^b^	Linearized standard error	95% Confidence Interval
Age	0.029	0.004*	[0.020–0.038]
Age squared	–0.0004	0.0003*	[–0.0005- −0.0002]
Last grade completed (*reference: university of higher*)			
Primary school or less	0.054	0.031^†^	[−0.007–0.115]
Secondary school	0.041	0.017**	[0.007–0.075]
High school	0.050	0.010*	[0.030–0.069]
Sexual identity (*gay = 1*)	0.049	0.010*	[0.028–0.069]
Number of sexual partners (last month)	0.004	0.001*	[0.001–0.006]
Condom use (last sexual intercourse)	0.049	0.012*	[0.026–0.072]
Sexual role (last sexual intercourse; *reference: insertive*)			
Receptive	0.054	0.013*	[0.029–0.079]
Insertive or receptive	0.070	0.013*	[0.046–0.095]
Region (*reference: Region 1*)			
Region 2	−0.108	0.016*	[–0.140– −0.076]
Region 3	−0.073	0.027*	[–0.125– −0.021]
Region 4	−0.081	0.017*	[–0.114– −0.048]
Region 5	0.018	0.039	[–0.059–0.095]

a Probit model for complex survey design, ^b^marginal effects reported; ^†^significant at 10%; **significant at 5%; *significant at 1%. F test (probit model): F(14–158) = 16.3, p<0.001.

The analytical sample for the probit model (n = 6,723) was smaller than the number of individuals who participated in the survey (n = 8,151), after excluding sex workers. We tested for statistical differences between the analytical sample and the subsample of individuals that was excluded from the analysis because they either did not have information on all covariates or did not accept the rapid HIV test. Statistical differences were as follows. We found that individuals who had information on all the variables were slightly younger (27.3 vs 28.2 years old), more likely to identify themselves as gay/homosexual (73.0% vs 68.6%) and reported lower rates of condom use (73.4% vs 78.4).

## Discussion

We estimated the HIV prevalence in 24 cities representative of the 5 regions in which the country was divided for program implementation purposes. The overall prevalence of 16.9% was significantly higher than expected given previous reports of around 10% [13]. However, to our knowledge, this is the first nationally representative survey among MSM that measures HIV prevalence in Mexico. Previous national estimates are mostly based on non-representative, small sample size surveys [14]. Unfortunately, this seems to be the case in many other countries in the region. Our results are more consistent with those recently reported by the testing program of an HIV clinic located in Mexico City which found an HIV prevalence of around 18% among a self-selected sample of 803 MSM in 2011 [25] and also more consistent with a recent report from Brazil, where researchers found a prevalence among MSM of 14% in a survey conducted in 10 cities in that country [3].

Hence, our results suggest that previous reports might have underestimated HIV prevalence among MSM in Mexico, or that HIV prevalence has been increasing in recent years whereby this survey captures the current state of such a trend. Obviously, a combination of both could also be happening. More studies with adequate design, sampling frames and sampling sizes are crucially important to address this question and adequately monitor the dynamics of HIV prevalence and of sexual risk behaviors among MSM. Regardless of whether the prevalence was underestimated or it has been growing, it is clear that there is an urgent need to properly design, target and implement HIV prevention strategies for MSM in Mexico. To the extent that our results in Mexico reflect the current situation in other LAC countries, this conclusion might be relevant for the entire region. Another conclusion that stems from our results has to do with the epidemiological surveillance systems in the countries of the region. Better-integrated monitoring systems linked to HIV testing programs and campaigns are needed in order to have a much better sense of the dimension and dynamics of the HIV epidemic.

Our results suggest that the risk of HIV infection increases with age, with the number of sexual partners and among those with a receptive sexual role. On the other hand, the risk decreases with education. All these predictors of HIV were expected and are consistent with our knowledge of the HIV epidemic and the risk factors associated with it. Less expected was the result that self-reported condom use is associated with higher risk of getting an HIV positive result. There are many possible reasons behind these results, one of them being that if individuals use condom more often with causal partners compared to stable partners, condom use may reflect more risky behaviors. In a simple probit regression (results not shown here), we found that a higher number of partners in the last month was associated with a higher probability of reporting using condom in the last sexual intercourse. At the same time, we find that more sexual partners predict a higher risk of an HIV-positive result. Another possible reason is that individuals at higher risk of infection may be more likely to over-report consistent condom use.

Participants in this survey were highly educated, as more than 70% reported having completed high school or higher education compared to 33% among urban men in the country. Although the survey did not reach marginalized populations, 5% of the sample had primary or no education and 4.5% reported speaking an indigenous language. The high education profile of the MSM surveyed is consistent with the high educational and economic profile of HIV-positive men in Mexico City [(unpublished paper, 2012)]. It should also be highlighted though, that very poor populations were probably under-represented or not represented at all in the survey. As described above, non-referred, marginal sites in which individuals with lower socioeconomic status may attend were not mapped.

The results of this study are representative of MSM who gather in the types of places included in our sampling frame. The most marginal, poor and possibly high-risk sites, not referred by key informants or owners, are not represented in the study. In that sense, our results should probably be read as a lower estimate of the HIV prevalence among MSM who meet in these types of places to find sexual partners. On the other hand, the highly known and referred sites included in the sampling frame of this survey are representative of those where a high proportion of the MSM population meet, and therefore probably the most relevant for most of the HIV transmission in the country.

Furthermore, the survey is representative of MSM and not of subgroups such as bisexual men, gay/homosexual or transgender women. We recognize that MSM is a vague and heterogeneous classification that only identifies sexual practices but not sexual identities but it is also a very relevant definition for prevention purposes, which is the main focus of this study.

Our findings clearly highlight the need for effective prevention efforts targeted at high-risk populations in order to control and reduce the incidence of HIV among MSM in Mexico. According the Foundation for AIDS Research, Mexico was reaching less than 20% of MSM for prevention programs [26], which is extremely low given the findings of our paper showing a high HIV prevalence and little knowledge of HIV status among those with positive results. Given the current knowledge on the effectiveness of antiretroviral treatment as prevention, one particularly important activity to implement is testing among MSM through outreach campaigns. According to the results of our survey, only 31.6% [95% c.i.: 27.5%–35.6%] of those with an HIV-positive result were aware of their status. There is a great need for testing among this population, however they are not demanding these services. The gap between the potential demand for HIV testing and the little actual demand is a problem that needs to be addressed with innovative, effective methods to increase the coverage of testing services and linkage to care. For example, in a study conducted in Malawi in 2004, researchers found that with very small incentives, actual demand for HIV testing significantly increased [27]. Similar efforts need to be investigated and implemented in Mexico and the LAC region. Furthermore, serious efforts need to be made to improve linkage between testing and treatment. According to a recent study in the region “late presenters” – those individuals who after becoming aware of their HIV-positive status do not show up for treatment until a year later (on average) – accounted for 50% of the total cases of late treatment initiation in an HIV clinic in Mexico between 2000 and 2010 [11]. In a country like Mexico, with a very strong, universal access ART program, timely diagnosis of HIV and linkage to care should receive priority and strategic attention.
